# Supplementary material for: Rapid turnover of life-cycle-related genes in the brown algae
Source: Genome Biol. 2019 Feb 14;20:35. doi: 10.1186/s13059-019-1630-6 (PMC6374913; doi:10.1186/s13059-019-1630-6)
Supplement: Supplementary file 3 — Protein alignments, corresponding Gblocks html file and CDS sequences for the lineage conserved gametophyte-biased genes. (ZIP 2109 kb) [file 13059_2019_1630_MOESM3_ESM.zip › Con_GA_alignments/Ec-05_002810.aa_ali.fasta-gb.htm]

Ec-05\_002810.aa.fasta.trans.aa\_ali.fasta


## Gblocks 0.91b Results

Processed file: **Ec-05\_002810.aa.fasta.trans.aa\_ali.fasta**  
Number of sequences: **2**  
Alignment assumed to be: **Protein**  
New number of positions: **612** (selected positions are underlined in blue)

```
                         10        20        30        40        50        60
                 =========+=========+=========+=========+=========+=========+
Ec-05_002810     MPPKAKDKKGKQEVLPVGIDYTRRPLASAEPCDARPPPRRELPVVSAHHAFEEWDDDALL
MSTRG.36946.1    ------------------------------------------------------------
                                                                             


                         70        80        90       100       110       120
                 =========+=========+=========+=========+=========+=========+
Ec-05_002810     EMEWGMVRAEPFEDKDGRSFLPARISARVARWVRPTELLTNETGEDGKEEHQQHSSGAVG
MSTRG.36946.1    ------------------------------------------------------------
                                                                             


                        130       140       150       160       170       180
                 =========+=========+=========+=========+=========+=========+
Ec-05_002810     GNGTASGVEKGKAGGGADKGKGKSPKGKDKGPSVEPVVLEDVAVDLQGNPLPRIFVPRAA
MSTRG.36946.1    ------------------------------------------------------------
                                                                             


                        190       200       210       220       230       240
                 =========+=========+=========+=========+=========+=========+
Ec-05_002810     IVANDSATDEGAQTLSQEEVDAPVASSDAPHAGDREVAKQSSVPNATLPRGFKTGWSESQ
MSTRG.36946.1    ------------------------------------------------------------
                                                                             


                        250       260       270       280       290       300
                 =========+=========+=========+=========+=========+=========+
Ec-05_002810     RRDIASLLCKYNQQQHQQQEHLPPETSGDAAATITEKETPPEAAVERHHREEEGTSGDWS
MSTRG.36946.1    ---MSSMLPSGKEQQQHQEDRPPEIAGGQPAGAATN--VPPEKSVESPLEGENNVLPPAV
                      ##############################    #############        


                        310       320       330       340       350       360
                 =========+=========+=========+=========+=========+=========+
Ec-05_002810     --KEPPAYLRDEDIATLGAPSGGDIDPAMTAIFKAIDQLSSTVSSTGCPLLWETIYPKTA
MSTRG.36946.1    GLDAPPSILGAVDVAALGAPGGEDIDHAMTAIFKAVGRLSPTISRSGCPLLWESIHPKTS
                     ########################################################


                        370       380       390       400       410       420
                 =========+=========+=========+=========+=========+=========+
Ec-05_002810     NEVPCYNPCGRYLIKLFAAGRWRRVEVDDSIPLDATGRPVIAASEDPLELWPLLLSKALY
MSTRG.36946.1    AEAPCYNPCGRYAVKLFAAGRWRKVEVDDSIPVDAARCPVIAASEDPAELWPILLSKALY
                 ############################################################


                        430       440       450       460       470       480
                 =========+=========+=========+=========+=========+=========+
Ec-05_002810     KLYRLGGYERVKESTNASA---------AAATAAAAAN-SVAPEEPSGSMSVTGGRDE--
MSTRG.36946.1    KLYRLGGYGIAQEAISTAASHSAVTVVTTVATDATVQNGPTAQDATETCDSLAGGVADGI
                 ###################                      ##############     


                        490       500       510       520       530       540
                 =========+=========+=========+=========+=========+=========+
Ec-05_002810     -----AGGGSVGGVDG-----EDGDERDRARFVAFVLHSLAGWVPS-------------I
MSTRG.36946.1    NGTAIGGDGDGGRVGDEDDVDECGDQAGRARFVAFVLHSLTGWIPSGASCPTPTSTSTST
                                      #########################              


                        550       560       570       580       590       600
                 =========+=========+=========+=========+=========+=========+
Ec-05_002810     TTGVSAEVALVHGMTPTLTPSDLRLPALPPVDEEAEAARERLEERRRRQALFLHKKRRPR
MSTRG.36946.1    PSVVSAEMALAQG-TPSLTSDDLLLPALPPVDEEEEAARERLEERKRRQALFLHKKRRPR
                    ########## ##############################################


                        610       620       630       640       650       660
                 =========+=========+=========+=========+=========+=========+
Ec-05_002810     KVERKVVSMEEYAEVDSARLRKMNAVSAKVLAGDRREVYVVCMPGEPARPVLAVCPQVPP
MSTRG.36946.1    KVERKVISAEERAEVDAARVRKMRAVAAKVLNGHRREVYMVCMPGVPACPVLAVCPAMPS
                 ############################################################


                        670       680       690       700       710       720
                 =========+=========+=========+=========+=========+=========+
Ec-05_002810     ADTTSVCQSG------TTTSSAFLKHQNLEPVGAESTVIATAGVGTSSRSLQADGSDQQP
MSTRG.36946.1    AIQASANSIGNPPSLAQGSTHLIPEQQPRQGSGVASEFVVSAGVGTSTSTIHAQEQQTSS
                 ##########                ##################################


                        730       740       750       760       770       780
                 =========+=========+=========+=========+=========+=========+
Ec-05_002810     HQPSSSP----------------LATMETMKDG----EGATLPLGFLLKWETP--TNSHQ
MSTRG.36946.1    LQPLASLSITSTTTPAGSAAEEEIANTRVTAAAEATREDENLPLGFLLKWEITPAAKLLQ
                 ######                               ##############        #


                        790       800       810       820       830       840
                 =========+=========+=========+=========+=========+=========+
Ec-05_002810     NGTESGQRSGAAEETVAAEPAEVIGEPESE-VVPRDMGAGVSD---PRDGEAKNRVLYGS
MSTRG.36946.1    VPTVSTSKNGSASTHADAASSSGLAEDQQEQQEPNGVSSSAEEGATTQDREGENRVLYGS
                 ##############################                  ############


                        850       860       870       880       890       900
                 =========+=========+=========+=========+=========+=========+
Ec-05_002810     GRRRRD--------HGNG-RNATDRSEATSPATSLSKPEYLWLLPRQIELAGGMVVRALT
MSTRG.36946.1    GGRRRGRGRGGGLDKDKGRQSAMDRATGASTSASLSSPAFLWLSPRQIELAGGTVVRALT
                 #####                #######################################


                        910       920       930       940       950       960
                 =========+=========+=========+=========+=========+=========+
Ec-05_002810     KVDKKAYADIQYHWKLATPSEVLDAPNSASEKTGSKE-TKNKAKTKTPAKDSKT-AKGKG
MSTRG.36946.1    KVDKTAYADVQYHWKLVIPPEKTDQSNTAGEKVAGKDGKANKAKGKHSEKDGKTSVKGQG
                 ####################################    ##############  ####


                        970       980       990      1000      1010      1020
                 =========+=========+=========+=========+=========+=========+
Ec-05_002810     TQARSTSGERLLSSSSSSSSALLAGENSTPEAERSASGTAIARGIPVPKGLEGMQARDPG
MSTRG.36946.1    TKAANIPGDGVP-----PP---STGENVDGETDGTLSGTAIAIGNVVPKGLEGRQARDPG
                 ########                ####################################


                       1030      1040      1050      1060      1070      1080
                 =========+=========+=========+=========+=========+=========+
Ec-05_002810     SPPPIFLVITPSPRSAPKITTTASSMRQSADRDAPDGEKTIAQEREPEPGDSKTENEMDS
MSTRG.36946.1    SPPAIFLLVTPSPRSEPK-------------R----------------------------
                 ##################                                          


                       1090      1100      1110      1120      1130      1140
                 =========+=========+=========+=========+=========+=========+
Ec-05_002810     HRRNEETGELLGEGPPPPTAAAGAVASAVSEMSRGGVVESPPEMSAGCGVQRSSGERLEE
MSTRG.36946.1    ------------------------------------------------------------
                                                                             


                       1150      1160      1170      1180      1190      1200
                 =========+=========+=========+=========+=========+=========+
Ec-05_002810     EEEAEEEEEAEVLMNLWADQPGADITTKKADWNASAMVALQEVLPGVPEGLHTSAESFKP
MSTRG.36946.1    ------------------------------------------------------------
                                                                             


                       1210      1220      1230      1240      1250      1260
                 =========+=========+=========+=========+=========+=========+
Ec-05_002810     VPGGRLIMRVKHDLEPNGLPFTAKSAKIRLVKGGAPRVFRVLLQPGLGCCIGFFCTHAIE
MSTRG.36946.1    ------------------------------------------------------------
                                                                             


                       1270      1280      1290      1300      1310      1320
                 =========+=========+=========+=========+=========+=========+
Ec-05_002810     TGDAGQVWEEAGYNQVTETGKYDGARPGEWKILFRRSFRVVGECPKPIGQPESSPVSADP
MSTRG.36946.1    ------------------------------------------------------------
                                                                             


                       1330      1340      1350      1360      1370      1380
                 =========+=========+=========+=========+=========+=========+
Ec-05_002810     SDGGAGSGGGSPESSIPETAPEGVVHEENAADTGIIAATGDSTENSSCRGQRLEEAEYLC
MSTRG.36946.1    ------------------------------------------------------------
                                                                             


                       1390      1400      1410      1420      1430      1440
                 =========+=========+=========+=========+=========+=========+
Ec-05_002810     FVRAILHLPDEESRPSVRLAFVDEDTGREMLSPSLHTNAVPLAPNKCGYSVLAAACGRAK
MSTRG.36946.1    ------------------------------------------------------------
                                                                             


                       1450      1460      1470      1480      1490      1500
                 =========+=========+=========+=========+=========+=========+
Ec-05_002810     AIPKGAWRLQLLSDRPLLPLKLRADGGGDGSDPSANSAKGVSVVSHPDGGTGKEAAHGGS
MSTRG.36946.1    AVPPGS------------------------------------------------------
                                                                             


                       1510      1520      1530      1540      1550      1560
                 =========+=========+=========+=========+=========+=========+
Ec-05_002810     ATTVVPCQERVVYGGVYVPNKYLRLFKDVLKCASPLPFAVRLRCSLPGICLRLLFLDAAT
MSTRG.36946.1    ------------------------------------------------------------
                                                                             


                       1570      1580      1590      1600      1610      1620
                 =========+=========+=========+=========+=========+=========+
Ec-05_002810     GETIRDIRGKHILQIDPSSLSTNEMPSKDKGKGGSTDKAAGAAAAAEQAALAAPKIIIEA
MSTRG.36946.1    ------------------------------------------------------------
                                                                             


                       1630      1640      1650      1660      1670      1680
                 =========+=========+=========+=========+=========+=========+
Ec-05_002810     ALDWEKMEVSPSLLSELPHYFQPTPEGMAHFCPPPVEIPSRNSSRPSTSSEEETQQCDKG
MSTRG.36946.1    ---------------------------------------------------ENKQQPHEG
                                                                    #########


                       1690      1700      1710      1720      1730      1740
                 =========+=========+=========+=========+=========+=========+
Ec-05_002810     AGGGGDISRSSCTGDGETTISTPSISAPTATETGASSLALVPGTELPDRSPEDCMKRNDH
MSTRG.36946.1    ARGP--------------------------------------------------------
                 ###                                                         


                       1750      1760      1770      1780      1790      1800
                 =========+=========+=========+=========+=========+=========+
Ec-05_002810     KSNPEQPVRWWLEIVGAGEVEMAHDMSRVDSENKEVESWEAATPGRADRAALLWTQRQAS
MSTRG.36946.1    ------------------------------------------------------------
                                                                             


                       1810      1820      1830      1840      1850      1860
                 =========+=========+=========+=========+=========+=========+
Ec-05_002810     KVASLGNGDRSLTSSSSPENVATSSPTTSSNVSVNPKPGEEGYEDWIKARAAAVGVPVTL
MSTRG.36946.1    ------------------------------------------------------------
                                                                             


                       1870      1880      1890      1900      1910      1920
                 =========+=========+=========+=========+=========+=========+
Ec-05_002810     ERERHASRLALLAMPATGAIFTPEPIAPSSGSPSLVVASLSSPPVPSALISDEEMKKRAD
MSTRG.36946.1    ------------------------------------------------------------
                                                                             


                       1930      1940      1950      1960      1970      1980
                 =========+=========+=========+=========+=========+=========+
Ec-05_002810     ERVALRQTTMEFEKKWEADRVDMVEAGMQEMAMQLEYIRGREESIKQARLPFLRDRETIV
MSTRG.36946.1    ------------------------------------------------------------
                                                                             


                       1990      2000      2010      2020
                 =========+=========+=========+=========+=
Ec-05_002810     QDLIAKMRARKEAVEEERAALEPTTKGGKTKQATKGKPGKK
MSTRG.36946.1    ------------------------------------REEDX
```

```
Parameters used
Minimum Number Of Sequences For A Conserved Position: 2
Minimum Number Of Sequences For A Flanking Position: 2
Maximum Number Of Contiguous Nonconserved Positions: 8
Minimum Length Of A Block: 10
Allowed Gap Positions: None
Use Similarity Matrices: Yes
```

```
Flank positions of the 16 selected block(s)
Flanks: [246  275]  [280  292]  [305  439]  [462  475]  [502  526]  [544  553]  [555  670]  [687  726]  [758  771]  [780  810]  [829  845]  [862  936]  [941  954]  [957  968]  [985  1038]  [1672  1683]  

New number of positions in Ec-05_002810.aa.fasta.trans.aa_ali.fasta-gb:  612  (30% of the original 2021 positions)
```
